# Special Issue “Fractalkine (CX3CL1) and Its Chemoattractant and Adhesion Molecule Properties in Health and Disease”

**DOI:** 10.3390/ijms262210899

**Published:** 2025-11-10

**Authors:** Dariusz Szukiewicz

**Affiliations:** Department of Biophysics, Physiology & Pathophysiology, Faculty of Health Sciences, Medical University of Warsaw, 02-004 Warsaw, Poland; dariusz.szukiewicz@wum.edu.pl

The discovery of the unique chemokine CX3CL1 (fractalkine, neurotactin) in 1997 by two independent research teams led by Bazan and Pan initiated a new chapter in the understanding of inflammation and cell-to-cell communication [1,2,3]. While other chemokines are typically soluble, fractalkine exhibits form duality, manifesting itself as a membrane-bound molecule that can also be shed into a soluble form [4]. Its duality of function corresponds to its form; the membrane-bound form acts as a powerful cell adhesion molecule that binds leukocytes to endothelial cells during the inflammatory response, and the soluble form, fractalkine, is a potent chemoattractant that guides leukocytes such as T cells and monocytes toward sites of inflammation [5,6]. This versatility continues to make it difficult for researchers to interpret research results, which sometimes indicate contradictory actions of fractalkine in various physiological and pathological processes, particularly in the brain, inflammation, and cancer [7,8,9,10].

This Special Issue, dedicated to fractalkine, aims to update and summarize the current state of knowledge regarding its importance in viral infections [11,12] and its involvement in the pathomechanisms of neurodegenerative diseases [13,14], complications of diabetes [15,16], cancer [5], and nonobstructive coronary artery disease (NO-CAD) [17]. Opinions regarding these issues presented in this editorial also consider existing controversies.

Recent studies have shown that fractalkine plays an important role in regulating the host immune response to viral infection [11]. Changes in the expression of CX3CL1 and its sole receptor, CX3CR1, are implicated in the pathomechanisms of human immunodeficiency virus (HIV), SARS-CoV-2, influenza virus, cytomegalovirus (CMV), and respiratory syncytial virus (RSV) infections [11,12,18,19]. In the case of RSV, CX3CR1 is a coreceptor for the virus and is expressed by airway epithelial cells and diverse immune cells; this is due to the presence of a highly conserved amino acid motif (CX3C motif) in the RSV G protein that enables binding to CX3CR1 and is also present in fractalkine [12,20]. Altered functioning of the CX3CR1-CX3CL1 axis and, consequently, an impaired host immune response should therefore be considered during the development of an efficient vaccine and specific pharmacological treatment.

Since its discovery, fractalkine has been extensively studied in relation to the nervous system, where it is naturally highly expressed. The CX3CL1–CX3CR1 interaction plays an important role in neuron–microglia signaling and, as a crucial modulator of microglial activation, has become a target for innovative therapies for neuroinflammation and neurodegenerative diseases [13,21,22,23]. One of the rationales for such actions may be the results of recent in vitro studies, which revealed a loss of neurofilaments and spheroids in a neuron–astrocyte–microglia coculture system after the administration of cerebrospinal fluid from patients with Alzheimer’s disease. The soluble form of fractalkine may contribute to this destabilization of neurofilaments, as well as to disturbances in cellular adhesion processes and intercellular contacts [14]. Nevertheless, despite numerous studies regarding the involvement of CX3CL1 and CX3CR1 in the progression of various neuroinflammatory and neurodegenerative diseases, especially Alzheimer’s and Parkinson’s diseases, unambiguous interpretation of the results is often impossible because of incompatible methodologies [13]. Moreover, depending on the clinical context, CX3CL1-CX3CR1 signaling may have neuroprotective effects by inhibiting microglial inflammatory processes or, conversely, maintaining/intensifying inflammation and neurotoxicity [16,24].

Diabetes mellitus (DM) is the most common metabolic disease in humans, and its prevalence is increasing worldwide in parallel with that of obesity [25]. A low-grade inflammatory response is typical in patients with DM, especially type 2 DM (T2DM), and plays a significant role in its development and complications [26,27,28]. Chemokine markers of inflammation in patients with DM associated with microvascular dysfunction include increased circulating levels of fractalkine, especially in individuals with concomitant obesity [29,30].

In patients with diabetic retinopathy, a typical long-term complication of diabetes, soluble fractalkine plays critical roles in retinal inflammation, angiogenesis, and neuroprotection, balancing tissue damage and repair [15]. Although elevated fractalkine concentrations accompany retinal inflammation, fractalkine deficiency exacerbates the severity of diabetic retinopathy. Furthermore, exogenous fractalkine has been shown to reduce inflammation, oxidative stress, and neuronal damage in states of fractalkine deficiency [31,32]. Preclinical evidence has therefore indicated the therapeutic potential of fractalkine, as modulating the state of microglial activation to ameliorate neovascularization may prove beneficial for managing diabetic retinopathy [15,33].

Another serious complication of diabetes is diabetic encephalopathy, which is characterized by cognitive impairment and motor dysfunction [34]. The crucial factor in this chronic neurological complication is increased pathological microglia activity, which is largely dependent on disturbed CX3CL1-CX3CR1 signaling [16,35]. Here, fractalkine signaling fits into the picture of the vicious cycle of disease created by microglia–neuron interactions during the course of neuroinflammation accompanying diabetic encephalopathy (Figure 1).

Taking the above into account, the goal of therapeutic management in patients with diabetic encephalopathy should be, in addition to optimal glycemic control, the promotion of neuroprotective effects or the inhibition of the neurotoxic effects of the CX3CL1-CX3CR1 signaling axis [16].

The role of fractalkine in cancer progression and its related potential in antineoplastic therapy remain unclear, especially given the presence of two forms of this chemokine. The chemoattractant properties of fractalkine can limit tumor growth by increasing the infiltration of immune cells, including T cells, natural killer cells, and activated dendritic cells [5,6,37]. However, studies have demonstrated that tumorigenesis with enhanced metastasis and invasion requires increased tumor expression of fractalkine or its receptor [5,6,37]. These discrepancies regarding the prognostic significance of CX3CL1-CX3CR1 axis activity in different cancers may indicate that the role of fractalkine in tumor biology cannot be limited to interactions with its cognate receptor [6]. Fractalkine interactions with only CX3CR1 may not cover the full spectrum of its pro- or antitumor immunoactivity [5].

Nonobstructive coronary artery disease (NO-CAD) is a heterogeneous group of conditions characterized by less than 50% narrowing in at least one major coronary artery with a fractional flow reserve (FFR) of ≤0.80, as observed by coronary angiography [17]. The results of recent studies indicate that fractalkine signaling may be an important factor in the pathogenesis and progression of NO-CAD, whose proinflammatory, proatherosclerotic, and microvascular-dysfunction-causing effects are well documented [17,38]. Moreover, promising preliminary results have shown that administering the CX3CR1 antagonist KAND567 to patients with ST-elevation myocardial infarction (STEMI) undergoing percutaneous coronary intervention (PCI) can prevent hyperinflammation, an adverse reaction of the immune system [17,39]. However, further research is needed to determine why the number of CX3CR1-expressing circulating effector T cells decreases substantially after reperfusion only in some patients [39].

## Figures and Tables

**Figure 1:**
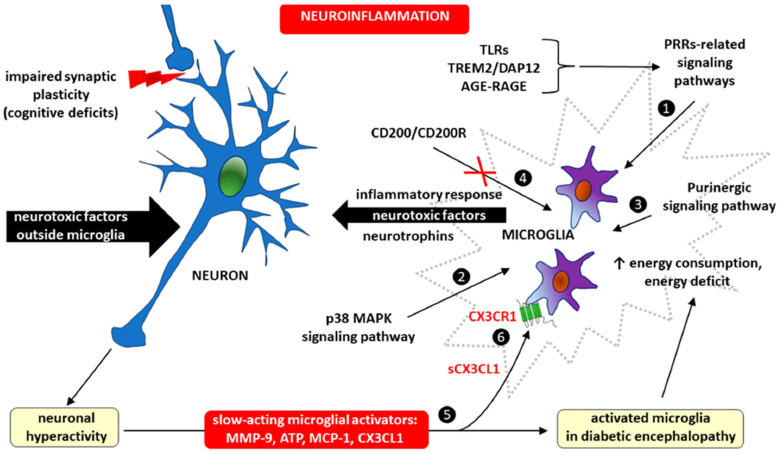
The importance of microglia and CX3CL1/CX3CR1 signaling in the vicious cycle of microglia–neuron interaction during neuroinflammation in diabetic encephalopathy. Adopted from [16]. Microglial activation in diabetic encephalopathy occurs as a result of the action of signaling pathways related to the following: ❶ pattern recognition receptors (PRRs), including Toll-like receptors (TLRs), triggering receptor expressed on myeloid cells 2/DNAX-activating protein of 12 kDa (TRM2/DAP12), and advanced glycation end product/receptor for advanced glycation end product (AGE-RAGE); ❷ the activation of p38 mitogen-activated protein kinases, a class of mitogen-activated protein kinases (MAPKs), p38 MAPK, and ❸ purinergic signaling. Microglial inflammation may also develop as a consequence of blocking the interaction of the immunomodulatory protein CD200 with its receptor CD200R (marked with crossed-out red lines) ❹, because the CD200/CD200R signaling induces immunosuppression due to the inhibition of macrophages, the induction of regulatory T cells, the switching of cytokine profiles from T helper-1 (Th1) to T helper-2 (Th2), the inhibition of tumor-specific T-cell immunity, and the induction of myeloid-derived suppressor cells (MDSCs) [36]. Neuronal hyperactivity in diabetic encephalopathy may be caused by both neurotoxic factors outside microglia and an inflammatory response within microglia. Neurotoxic agents increase the risk of cognitive deficits due to impaired synaptic plasticity. Whatever the cause, neuronal hyperactivity has a feedback-activating effect on microglia by releasing slow-acting microglial activators, such as matrix metalloproteinase-9 (MMP-9), adenosine triphosphate (ATP), monocyte chemoattractant protein-1 (MCP-1), and fractalkine (CX3CL1) ❺. The soluble form of CX3CL1 (sCX3CL1) has a proinflammatory effect by stimulating metabotropic CX3CR1 receptors expressed in microglial cells ❻.
